# Ras-dva1 small GTPase regulates telencephalon development in *Xenopus laevis* embryos by controlling Fgf8 and Agr signaling at the anterior border of the neural plate

**DOI:** 10.1242/bio.20147401

**Published:** 2014-02-25

**Authors:** Maria B. Tereshina, Galina V. Ermakova, Anastasiya S. Ivanova, Andrey G. Zaraisky

**Affiliations:** Shemyakin-Ovchinnikov Institute of Bioorganic Chemistry, Russian Academy of Sciences, Moscow 117997, Russia

**Keywords:** Forebrain development, Anterior neural border, Agr, Fgf8, FoxG1, Otx2, Ras-dva

## Abstract

We previously found that the small GTPase Ras-dva1 is essential for the telencephalic development in *Xenopus laevis* because Ras-dva1 controls the Fgf8-mediated induction of *FoxG1* expression, a key telencephalic regulator. In this report, we show, however, that *Ras-dva1* and *FoxG1* are expressed in different groups of cells; whereas *Ras-dva1* is expressed in the outer layer of the anterior neural fold, *FoxG1* and *Fgf8* are activated in the inner layer from which the telencephalon is derived. We resolve this paradox by demonstrating that Ras-dva1 is involved in the transduction of Fgf8 signal received by cells in the outer layer, which in turn send a feedback signal that stimulates *FoxG1* expression in the inner layer. We show that this feedback signal is transmitted by secreted Agr proteins, the expression of which is activated in the outer layer by mediation of Ras-dva1 and the homeodomain transcription factor Otx2. In turn, Agrs are essential for maintaining *Fgf8* and *FoxG1* expression in cells at the anterior neural plate border. Our finding reveals a novel feedback loop mechanism based on the exchange of Fgf8 and Agr signaling between neural and non-neural compartments at the anterior margin of the neural plate and demonstrates a key role of Ras-dva1 in this mechanism.

## INTRODUCTION

In vertebrate embryos, a stripe of cells located at the anterior neural plate border (ANB) is an important developmental organizer, which generates the Fgf8-based signal that regulates patterning of the presumptive rostral forebrain, including the telencephalon and the anterior part of the diencephalon (Cavodeassi and Houart, 2012).

Recently, we have shown that in the *Xenopus laevis* embryo, the small GTPase Ras-dva1, which is expressed near ANB, is necessary for the Fgf8-mediated expression of the telencephalic regulator *FoxG1* (previously named *BF1*) in cells at the anterior margin of the neural plate (Tereshina et al., 2006). This expression pattern indicates that Ras-dva1, which is a membrane-bound GTPase (Tereshina et al., 2007), might control the propagation of Fgf8 signal within these cells, composing the telencephalic rudiment. As we now demonstrate, however, *Ras-dva1* is not co-expressed with *FoxG1* in the presumptive telencephalic cells. The expression of *Ras-dva1* occurs exclusively in the non-neural cells in the presumptive cement and hatching glands, which compose the outer layer of the rostral part of the anterior neural fold and the adjacent non-neural ectoderm surrounding the neural fold at the anterior. Therefore, we hypothesized that Ras-dva1 might control the expression of *FoxG1* non-autonomously via regulation of some unknown signal sent by the non-neural cells to the adjacent cells in the presumptive telencephalon.

By using gain- and loss-of-function approaches, we tested this hypothesis and found that the presumptive telencephalic cells induce the expression of *Ras-dva1* through secreted Fgf8 in cells of the adjacent non-neural ectoderm. As a result, the Ras-dva1-mediated Fgf8 signal induces the expression of the homeobox gene *Otx2* in these cells. In turn, Otx2 activates genes encoding the Agr secreted factors, Xag and Xagr2, which belong to the superfamily of protein disulfide isomerases and supposedly to modulate protein folding, but also possess an independent signaling activity (Blassberg et al., 2011; Hatahet and Ruddock, 2009; Persson et al., 2005; Vanderlaag et al., 2010). We demonstrate that Xag and Xagr2 are required for the Fgf8-dependent activation of *FoxG1* expression in the presumptive telencephalic cells. An interruption in this signaling feedback loop at any step, and in particular downregulation of *Ras-dva1* expression, leads to severe malformations of the rostral forebrain, as well as abnormalities of the structures deriving from the non-neural anterior ectoderm.

## MATERIALS AND METHODS

### DNA constructs, synthetic mRNAs and morpholino oligonucleotides

All plasmids were described previously (Ermakova et al., 1999; Ermakova et al., 2007; Ivanova et al., 2013; Novoselov et al., 2003; Tereshina et al., 2006). Synthetic capped mRNA and anti-sense RNA dig-labeled probes for *in situ* hybridization were prepared by using mMessage Machine Kits (Ambion). RNA templates were purified by the RNeasy Mini Column Kit (QIAGEN). Embryos were injected at the 8- or 16-cell stages with 8 or 4 nl per blastomere of mRNA water solution, respectively. The mRNA concentrations were 50 pg/nl of *FGF8a* and 60 pg/nl of each *Agrs* and *Ras-dva1* mRNAs. The Morpholino Oligonucleotides (MO) were from Gene Tools LLC (see supplementary material Table S1 for MO sequences). All MOs were dissolved in RNAse-free water to a concentration of 0.4 mM, mixed before injection with Fluorescein Lysine Dextran (FLD) (Invitrogen, 40 kDa, 5 mg/ml) tracer and injected into blastomeres at volumes of either 4 nl (at 16-cell stage) or 8 nl (at 8-cell stage). In case when the mixture of *Xag* and *Xagr2* MO was injected, the final concentration of each MO was 0.2 mM.

To construct templates for testing the efficiency of *Xag* and *Xagr2* MOs, cDNA fragments containing the MO target sites along with open reading frames of *Xag2* and *Xagr2A* were obtained by PCR, cloned into the Evrogen pTagRFP-N vector (cat. no. FP142) by *EcoRI* and *AgeI* upstream and in-frame of the TagRFP cDNA, followed by recloning of the *Xag2-TagRFP* and *Xagr2-TagRFP* cassettes (excised by *Eco*RI and blunted *Not*I) into *Eco*RI and blunted *XhoI* sites of the pCS2+ vector. Capped mRNA encoding Xag2-TagRFP and Xagr2-TagRFP was synthesized with SP6 Message Machine Kit (Ambion) using the obtained plasmids cut by *Not*I. The resulting mRNA was injected into dorsal blastomeres of 8-cell embryos (100 pg/blastomere) either alone or in a mixture with the corresponding MO (8 nl of 0.4 mM water solution) (supplementary material Fig. S1A). The injected embryos were collected at the midneurula stage and analyzed for the presence of Xag2-TagRFP and Xagr2-TagRFP by Western blotting with Evrogen tRFP antibody (cat. no. AB233) as described (Bayramov et al., 2011). As a result, the complete suppression of *Xag2-TagRFP* and *Xagr2-TagRFP* mRNA translation was observed in embryos co-injected with the corresponding anti-sense MO (supplementary material Fig. S1). In contrast, no inhibition was observed if *mis-Xag*, *mis-Xagr2* or a standard control MO was co-injected.

### Transgenic embryos and *in situ* hybridization

To generate constructs expressing *Xanf1* or *dnRas-dva1* under the control of the *Xenopus laevis Xag2* promoter, cDNA fragments encoding these proteins were obtained by PCR and sub-cloned together with a 1.2 kb fragment of *Xag2* promoter into the *pRx-GFP-pCA-RFP* double-cassette vector (a gift from R. Grainger) in place of the *Rx-GFP* cassette. Transgenic embryos bearing the resulting constructs, *pXag2-Xanf1-pCA-RFP* and *pXag2-DNRas-dva1-pCA-RFP*, were generated as described (Martynova et al., 2004; Offield et al., 2000); transgenic embryos were selected either by performing in situ hybridization with the probe of interest mixed with a *RFP* probe or by observing red fluorescence in the skeletal muscles of the tadpoles (Ermakova et al., 2007).

Whole-mount in situ hybridization was performed as described (Harland, 1991), mainly with dig- and in case of the double in situ also with fluorescein-labeled probes. For in situ hybridization on left and right halves of individual embryos, the embryos were dissected along the medial sagittal plane and processed as described (Tereshina et al., 2006). For in situ hybridization of tissue sections, embryos were first embedded in 4% agarose and then dissected on a vibratome into 20 µm serial, sagittal sections; the central pair of adjacent sections from each embryo were selected and processed individually for in situ hybridization as described (Harland, 1991).

## RESULTS

### Ras-dva1 regulates rostral forebrain development via a non-autonomous mechanism

**Fig. 1.**
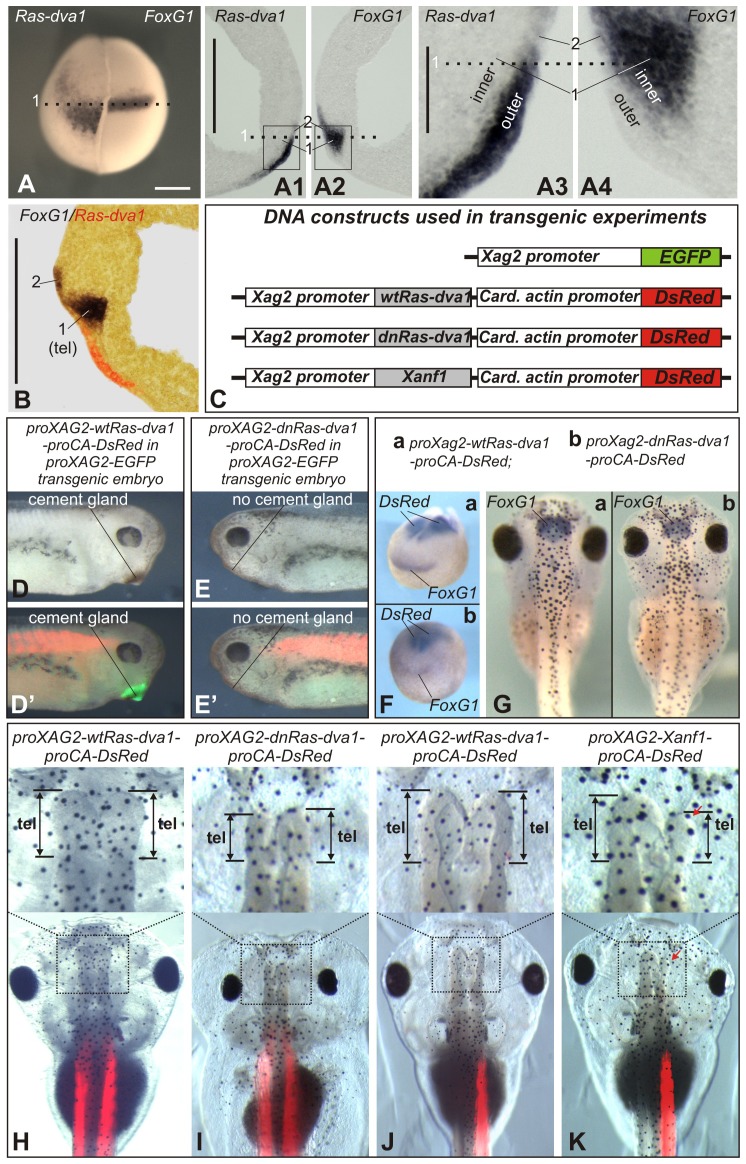
Ras-dva1 is expressed in the non-neural anterior ectoderm and regulates forebrain development by a cell non-autonomous mechanism. (A) In situ hybridization with dig-labeled probes to *Ras-dva1* and *FoxG1* on the left and right halves of the same embryo. Upon completing the in situ hybridization procedure, the two halves of the embryo were stacked together and photographed from the anterior with the dorsal side upward. The dotted line corresponds to the dotted lines in panels A1–A4. (A1,A2) Adjacent vibratome medial sagittal sections of the same embryo were hybridized separately with *Ras-dva1* (left section) or *FoxG1* (right section) probe. Anterior sides face each other, dorsal sides up. “1” indicates region of the inner layer in which only *FoxG1* is expressed. “2” indicates region of the outer layer in which *Ras-dva1* and *FoxG1* are co-expressed. (A3,A4) Enlarged images of fragments squared in panels A1 and A2. (B) In situ hybridization with dig- and fluorescein-labeled probes to *Ras-dva1* and *FoxG1* on the vibratom medial sagittal section of the midneurula (stage 15) embryo. (C) Schemas of DNA constructs used to generate transgenic embryos shown in panels C–J. (D–E′) Whereas no cement gland inhibition is seen in control embryo of transgenic line bearing proXag2-EGFP construct and transfected by *proXag2-wtRas-dva1-proCA-DsRed* (D,D′), the embryo of the same line but transfected by *proXAG2-dnRas-dva1-proCA-DsRed* construct has no cement gland (E,E′). (F) No inhibition of *FoxG1* expression is seen in the early neurula embryo bearing the control transgene (*proXag2-wtRas-dva1-proCA-DsRed*) (a). In contrast, a decrease of *FoxG1* expression is observed in the embryo transfected with *proXAG2-dnRas-dva1-proCA-DsRed* (b). Whole-mount in situ hybridization with probes to both *FoxG1* and *DsRed*. (G) Transgenic tadpole bearing the control *proXag2-wtRas-dva1-proCA-DsRed* construct has normal sized telencephalon marked by *FoxG1* expression. At the same time, a reduction of the telencephalon and *FoxG1* is seen in embryo bearing *proXAG2-dnRas-dva1-proCA-DsRed* construct. Transgenic tadpoles were selected by revealing DsRed fluorescence and hybridized in whole-mount with the probe to *FoxG1*. (H–K) The telencephalons (upper row) and whole heads (bottom row) of the 5-day tadpoles bearing transgenic constructs indicated on the top. Scale bars: 200 µm (A–A2,B), 40 µm (A3,A4).

When analyzing *FoxG1* and *Ras-dva1* expression in whole mount embryos, one may conclude that these genes are co-expressed in same anterior neural fold cells (Fig. 1A). However, we have found that this conclusion is not correct because these genes are mostly expressed in different groups of cells. Namely, beginning from the gastrula stage and till at least tailbud stage, *Ras-dva1* is intensively expressed exclusively in cells in the outer layer of the non-neural ectoderm (Fig. 1A1,A3,B; supplementary material Fig. S1B1,E1), which during neurulation partially overlaps the rostral part of the underlying inner layer of the anterior neural fold, where *FoxG1* is expressed and from which the telencephalon derives (zone 1 in Fig. 1A1–A4,B; zone 1 in supplementary material Fig. S1I,K1,K3). In addition, lower expression of *FoxG1* and *Ras-dva1* was observed in a scattered stripe of the outer layer cells, located posterior to cells intensively expressing *Ras-dva1* (zone 2 in Fig. 1A1–A4,B; zone 2 in supplementary material Fig. S1I,K1,K3). Importantly, these outer cells, in which both *FoxG1* and *Ras-dva1* are expressed, further give rise to the diencephalon (supplementary material Fig. S1K5), but not to the telencephalon (G. Eagleson, personal communication; see also Eagleson et al., 1995; Eagleson and Harris, 1990; Eagleson et al., 1986). Thus, *Ras-dva1* is not co-expressed with *FoxG1* directly in the telencephalic primordium.

In contrast to the spatial complementarity of the *Ras-dva1* and *FoxG1* expression domains, the activity of *Ras-dva1* appears to be critical for *FoxG1* expression and telencephalon development. Thus, injections of antisense MO or an mRNA encoding the dominant-negative variant of Ras-dva1 (dnRas-dva1) led to a reduction in the telencephalon, eyes and other anterior structures (Tereshina et al., 2006). These results indicate that Ras-dva1 may regulate *FoxG1* expression and forebrain development by a non-autonomous mechanism.

To more clearly demonstrate the non-autonomous character of this mechanism, we precisely inhibited *Ras-dva1* expression in cells of the outer layer. To this end, we created transgenic embryos in which either wild-type *Ras-dva1*, its dominant-negative mutant *dnRas-dva1* (Tereshina et al., 2006), or the natural inhibitor of *Ras-dva1*, the homeodomain transcriptional repressor Xanf1 (Ermakova et al., 1999; Tereshina et al., 2006), was expressed under the control of a 1.2 kb fragment of *Xag2* promoter (Fig. 1C). As we have shown previously, this promoter fragment is sufficient to specifically target the expression of the fluorescent reporter to cells in the presumptive hatching and cement gland domains, located in the outer layer of the non-neural anterior ectoderm, in which *Ras-dva1* is expressed (Ivanova et al., 2013; Serebrovskaya et al., 2011).

The DNA cassette, composed of the *Xag2* promoter attached to either wild-type *Ras-dva1*, *dnRas-dva1* or *Xanf1* cDNA, was a part of the double-cassette vector; the second cassette contains the DsRed red fluorescent protein cDNA (Matz et al., 1999) under the control of the *Cardiac Actin* promoter (Fig. 1C). The *DsRed* expression cassette helped us to distinguish transgenic embryos from the non-transgenic ones by detecting *DsRed* RNA (at early stages) or by fluorescence (at late stages).

When transgenic embryos were obtained, we found that approximately 40% and 50% (28 and 35) of embryos bearing *proXag2-dnRas-dva1-proCA-DsRed* and *proXag2-Xanf1-proCA-DsRed* constructs, respectively, had no cement glands (Fig. 1D–E′; supplementary material Fig. S2A–C). In addition, and in contrast to transgenic embryos expressing the wild-type *Ras-dva1*, approximately 45% of those expressing transgenic *dnRas-dva1* and 60% expressing *Xanf1* (*n* = 24 and 21, respectively) demonstrated inhibition of expression of the forebrain regulator *FoxG1* (Fig. 1F; supplementary material Fig. S2D). A reduction in the *FoxG1* expression zone was also observed in tadpoles developed from these embryos (10/15 and 12/17) (Fig. 1G; supplementary material Fig. S2E). Consistently, transgenic tadpoles bearing the *proXag2-dnRas-dva1-proCA-DsRed* and *proXag2-Xanf1-proCA-DsRed* constructs had reduced telencephalons and eyes (21/30 and 26/32), i.e. the forebrain abnormalities resembling those observed earlier in embryos injected with *dnRas-dva1* mRNA or *Ras-dva1* MO (Tereshina et al., 2006) (Fig. 1H–K) These effects were especially evident in accidentally appearing embryos, having transgenic constructs in cells of only left or right side of the body. In these embryos, non-transgenic halves may serve as internal controls (Fig. 1J,K). Taken together, these results confirm the non-autonomous character of the mechanism by which Ras-dva1 regulates forebrain development.

### Ras-dva1 mediates the Fgf8-dependent induction of *Agrs* expression in the outer layer of the non-neural anterior ectoderm

**Fig. 2.**
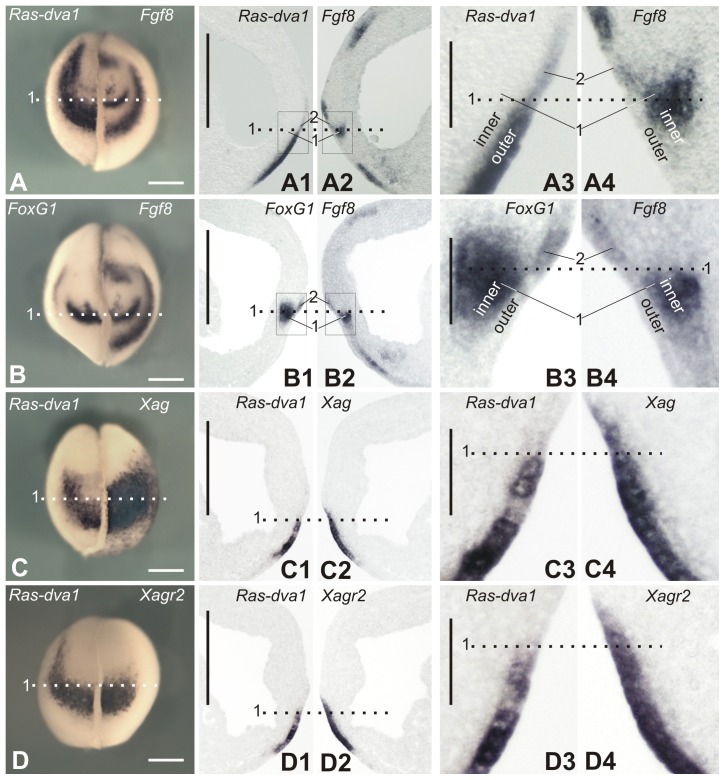
Pairwise comparison of expression patterns of genes expressed in the anterior ectoderm at the midneurula stage. (A–D) Whole-mount in situ hybridization with probes to transcripts of the indicated pairs of genes on the left and right halves of individual embryos as it is described in Fig. 1A. (A1–D1,A2–D2) In situ hybridization on adjacent vibratome sagittal sections of individual embryos with probes to indicated pairs of transcripts. Note that panels C1,D1,C2,D2 show results of hybridization made on two pairs of adjacent sections of the same embryo. (A3–D3,A4–D4) Enlarged images of fragments framed in panels A1–D1 and A2–D2. For abbreviations, see Fig. 1A–A4. Scale bars: 200 µm (A–D2), 40 µm (A3–D4).

As we have demonstrated previously, downregulation of *Ras-dva1* can block the induction of *FoxG1* expression elicited by ectopic Fgf8a (Tereshina et al., 2006). Together with the present data suggesting the non-autonomous character of the Ras-dva1-mediated stimulation of *FoxG1* expression in the telencephalic primordium, this result indicates that the role of Ras-dva1 in this process may be in the transmission of the Fgf8 signal within cells in the outer layer, thereby programming them to initiate some feedback signal, which in turn induces cells in the inner layer to express *FoxG1*. Consistently, similarly to *FoxG1*, *Fgf8* is expressed in cells of the inner layer of the anterior neural fold from which the telencephalon derives (zone 1 in Fig. 2A2,A4,B2,B4). In contrast to *FoxG1*, however, a low *Fgf8* expression can be also seen in the inner layer, in a region entirely underlying the territory of the cement gland placode in the outer layer, in which *Ras-dva1* is expressed most intensively (compare Fig. 2A2 with Fig. 2B3). Similarly to *FoxG1*, low *Fgf8* expression is revealed in non-telencephalic cells of outer layer, in which *Ras-dva1* is also expressed (zone 2 in Fig. 2A2,A4,B2,B4; zone 2 in supplementary material Fig. S1J,K2,K4).

The hypothetical feed-back signal produced by the outer layer cells could be transmitted by some factor(s) secreted by these cells. We supposed that the role of such factors could play secreted Agr proteins, which belong to the superfamily of protein disulfide isomerases (Persson et al., 2005). The reasoning for this hypothesis is that first, *Agrs* are expressed in a similar region of the anterior ectoderm as *Ras-dva1*, and second, at least one of the *Xenopus* Agrs, Xag2, was shown to induce anterior neural fate in the embryonic ectoderm through a Fgf signaling-dependent manner (Aberger et al., 1998).

Those of *Agrs* that are expressed during gastrulation and neurulation are represented in the *Xenopus laevis* genome by two pairs of very homologous pseudoalleles, *Xag1/Xag2* and *Xagr2a/Xagr2b* (further referred to as *Xag* and *Xagr2*, respectively) (Ivanova et al., 2013). As we have identified by in situ hybridization, these genes are indeed co-expressed with *Ras-dva1* in cells in the outer layer of the anterior ectoderm, which further give rise to the cement and hatching glands (Fig. 2C–C6,D–D6; supplementary material Fig. S1B2,F,H1). Importantly, although *Agrs*, like *Ras-dva1*, are co-expressed with *FoxG1* and *Fgf8* in the outer layer of the anterior neural ridge, no expression of these genes were detected in cells of the telencephalic primordium located in the inner layer of the ridge (supplementary material Fig. S1H2). Thus, the secreted protein products of *Agrs* could potentially play a role as the proposed signaling factors regulated by Ras-dva1.

**Fig. 3.**
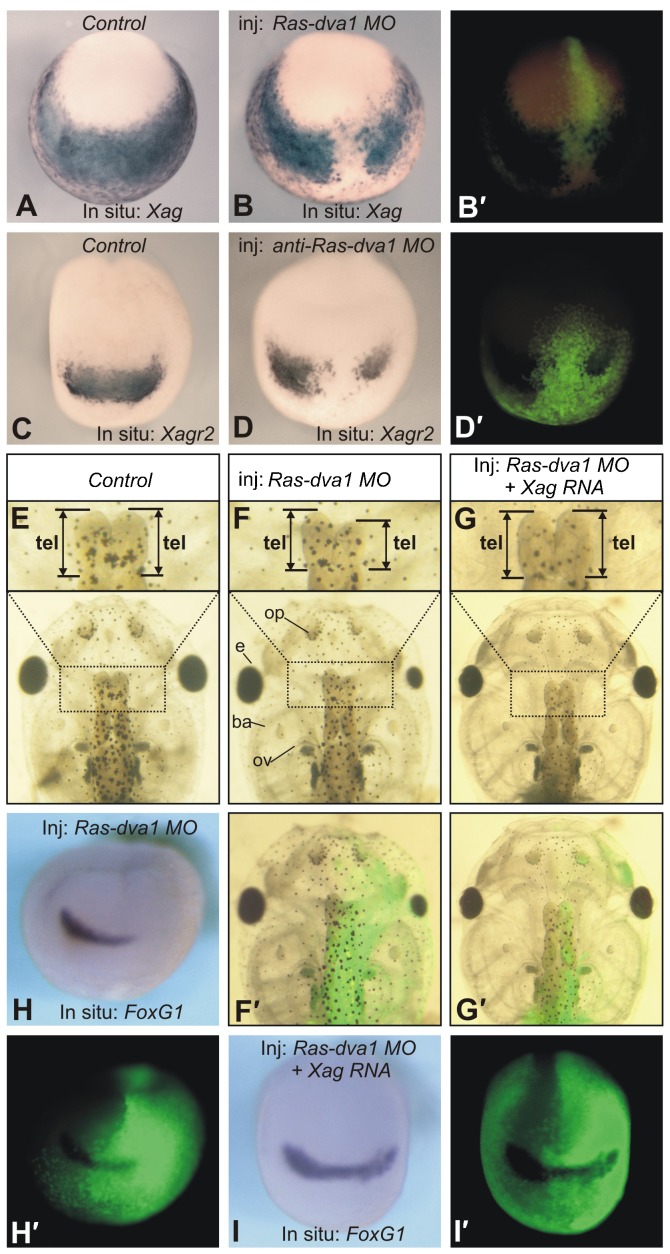
Inhibition of *Ras-dva1* mRNA translation by the *Ras-dva1* morpholino elicits the downregulation of *Agrs* and a reduction of the forebrain. (A,C) Whole-mount in situ hybridization of midneurula control embryos with dig-labeled probes to *Xag* and *Xagr2*, respectively. Anterior view with dorsal side upward. (B,B′,D,D′) Whole-mount in situ hybridization of the *Ras-dva1* MO-injected midneurula embryos with dig-labeled probes to *Xag* and *Xagr2*, respectively. Fluorescent images in panels B′ and D′ demonstrate distribution of cell clones containing the co-injected FLD fluorescent tracer. (E) The telencephalon (upper row) and whole head of the control 5-day tadpole. Dorsal view, anterior to the top. (F,F′) The telencephalon (upper row) and whole head of the 5-day tadpole developed from the embryos injected with *Ras-dva1* MO into the right dorsal blastomere at the 8-cell stage. Note the reduced telencephalon and eye on the injected side. The fluorescent image in panel F demonstrates the distribution of cell clones containing the co-injected FLD fluorescent tracer. (G,G′) Rescue of the *Ras-dva1* MO-induced abnormalities by the co-injection of *Ras-dva1* mRNA. Note the normal telencephalon and eye on the injected side (see distribution of the injected cells in panel G′). (H,H′) Whole-mount in situ hybridization of the *Ras-dva1* MO-injected midneurula embryos with dig-labeled probes to *FoxG1*. Note the inhibition of *FoxG1* expression on the injected side. See the distribution of cell clones containing the co-injected FLD fluorescent tracer in panel H′. (I,I′) Rescue of the *Ras-dva1* MO-induced inhibition of *FoxG1* expression by the co-injection of *Ras-dva1* mRNA. See the distribution of cell clones containing the co-injected FLD fluorescent tracer in panel I′.

To verify whether Ras-dva1 activity is necessary to induce *Agrs* expression, we first tested whether downregulation of *Ras-dva1* could influence their expression. When embryos were injected with *Ras-dva1* MO, a severe downregulation of *Xag*, *Xagr2* and *FoxG1* at the midneurula stage (20/23, 25/28, 23/25) and a reduction of the telencephalon and eyes of tadpoles (31/39) were observed (Fig. 3A,B,C,D,E,F,H). In contrast, no abnormalities were seen when the control misRas-dva1 MO was injected (see supplementary material Fig. S3 for results of injections of this and other control MO). Importantly, a rescue of these abnormalities, including normalization of the expression pattern of *FoxG1* and a restoration of the wild-type forebrain phenotype, was observed when *Ras-dva1* MO was co-injected with either *Xagr2* or *Xag* mRNA (here and below designates *Xagr2A* and equimolar mixture of *Xag1* and *Xag2*, respectively) (22/35 and 23/34) (Fig. 3G,I and data not shown). This result indicates that Agrs is downstream of Ras-dva1 in the mechanism regulating the forebrain development.

**Fig. 4.**
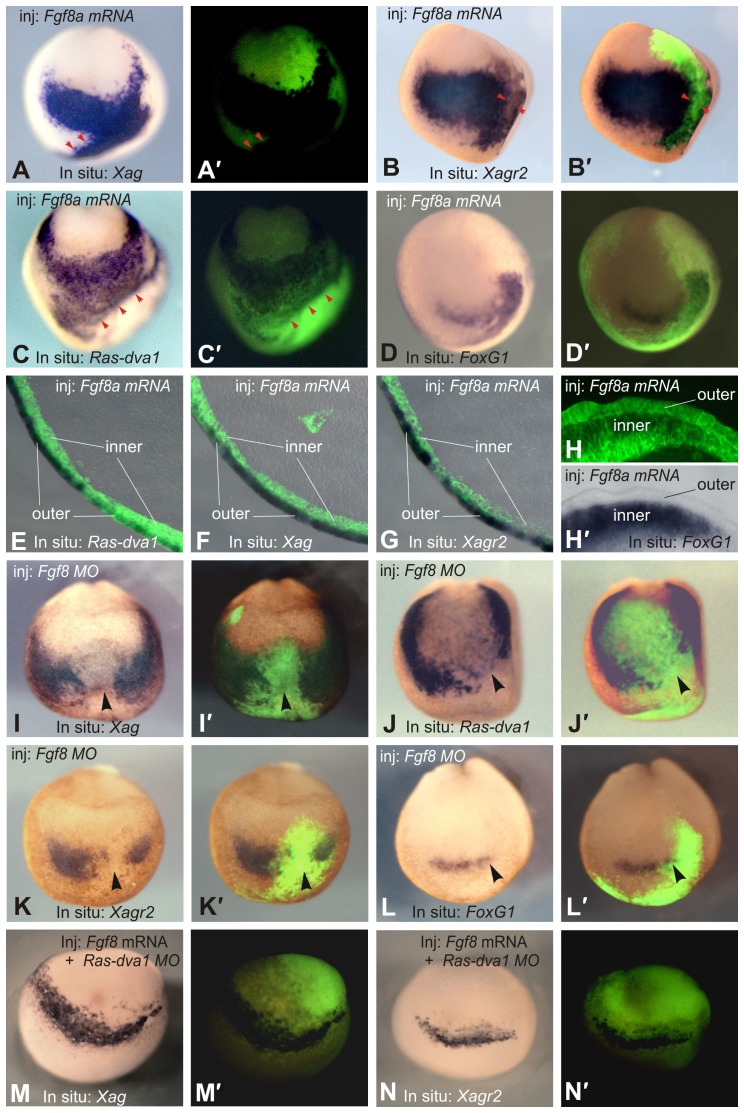
Fgf8 regulates the expression of *FoxG1*, *Ras-dva1*, *Xag* and *Xagr2*. (A–D) Injections of *Fgf8a* mRNA elicit ectopic expression of *FoxG1*, *Ras-dva1*, *Xag* and *Xagr2* in the anterior ectoderm of the midneurula stage embryos. *Fgf8a* mRNA mixed with FLD tracer was injected at concentration of 20 pg/blastomere in a pair of adjacent animal dorsal and ventral blastomeres on the left sides of 16- to 32-cell stage embryos. Whole-mount in situ hybridization was performed at the midneurula stage. Anterior view with the dorsal side upward. (A′–D′) Overlays of the white light and fluorescent images of embryos shown in panels A–D. Red arrowheads in panels A and C indicate the borders of ectopic expression, which correspond to the borders of cell clones with strong fluorescence. (E–H) Vibratome sections of embryos injected with Fgf8a mRNA. Note that whereas *Ras-dva1*, *Xag* and *Xagr2* are activated only in the outer layer of the ectoderm (E–G, overlays of bright light and fluorescent images), ectopic *FoxG1* is induced exclusively in the inner layer (H,H′). (I–L) Injection of *Fgf8a* MO leads to inhibition of *FoxG1*, *Ras-dva1*, *Xag* and *Xagr2* expression. An *Fgf8a* MO (1–2 pmol/blastomere) was injected with a FLD tracer in a pair of adjacent animal dorsal and ventral blastomeres on the left sides of 16- to 32-cell stage embryos. (M,N) Co-injection of *Fgf8* mRNA is unable to rescue inhibition of *Xag* and *Xagr2* expression elicited by *Ras-dva1* MO. The black arrowheads indicate sites of expression inhibition.

If the Ras-dva1-mediated expression of *Agrs* in the non-neural cells is activated by Fgf8 signaling, then disturbances in this signaling might also influence *Agrs* expression. To test this prediction, we examined whether Fgf8 can activate expression of *Xag* and *Xagr2* in the anterior ectoderm of midneurula embryos injected with *Fgf8a* mRNA. Indeed, we observed strong ectopic induction of *Xag* and *Xagr2* expression in cells in the non-neural ectoderm (25/26 and 23/24) (Fig. 4A,B). Importantly, the expression of *Ras-dva1* and *FoxG1* appears to also be ectopically activated, confirming involvement of all four genes in the same regulatory pathway (Fig. 4C,D). Remarkably, in contrast to the ectopically induced expression of *FoxG1*, the expression of *Agrs* and *Ras-dva1* was observed not in *Fgf8* expressing cells but in cells at the direct periphery of the *Fgf8* expressing cells (arrowheads in Fig. 4B,C). Interestingly, as one may see in the histological sectioning of these embryos, whereas *Ras-dva1*, *Xag* and *Xagr2* are activated only in the outer layer of the ectoderm, ectopic FoxG1 is induced exclusively in the inner layer (Fig. 4E–H). These results also confirmed that *FoxG1* is expressed in relation to *Ras-dva1*, *Xag* and *Xagr2* in a spatially complementary pattern.

Conversely, we observed inhibition of *Xag*, *Xagr2*, *Ras-dva1* and *FoxG1* expression when translation of *Fgf8* mRNA was suppressed by injection of Fgf8 MO in the left or right pair of adjacent dorsal and ventral animal blastomeres at the 16- to 32-cell stage (the presumptive left or right half of the anterior ectoderm) (23/26, 24/27, 22/23 and 20/26, respectively) (Fig. 4I–L).

These results corroborate our working hypothesis predicting that Fgf8-dependent induction of *Agrs* expression is mediated by the Ras-dva1 activity. Additionally, these experiments revealed that the expression of *Ras-dva1* itself is under the control of Fgf8 signaling.

To confirm that Ras-dva1 mediates the induction of *Agrs* expression by Fgf8, we investigated whether downregulation of Ras-dva1 can interrupt induction of the ectopic expression of *Agrs* by Fgf8. Indeed, most embryos injected with a mixture of *Fgf8* mRNA and *Ras-dva1* MO did not demonstrate ectopic expression of *Xag* and *Xagr2* (24/25 and 23/27). Moreover, we observed partial downregulation of *Xag* expression in the injected side of some of the latter embryos (14/25 and 18/27) (Fig. 4M,N).

Based on these data, we concluded that the Fgf8 signal produced by cells of the anterior neural plate is essential for the induction of *Agrs* and *Ras-dva1* expression in cells in the outer layer of the adjacent non-neural ectoderm, and the activity of Ras-dva1 within the latter cells is crucial for this induction.

### Downregulation of *Agrs* elicits abnormalities similar to those observed in embryos with inhibited Ras-dva1 function

Our experiments described above demonstrate that *Xag* and *Xagr2* are located downstream of Fgf8 and Ras-dva1 in the signaling pathway that regulates *FoxG1* expression. This result indicates that in normal development Agr proteins produced by cells in the non-neural ectoderm could be responsible for stimulating *FoxG1* expression in cells of the presumptive telencephalon. To verify this prediction, loss-of-function experiments were performed by injecting *Xag* and *Xagr2* MO.

**Fig. 5.**
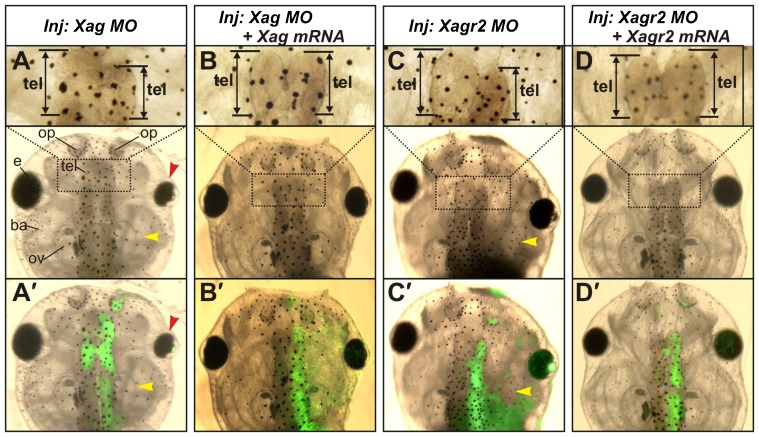
Inhibition of *Xag* and *Xagr2* mRNA translation by anti-sense morpholinos elicits brain abnormalities similar to those observed when *Ras-dva1* and *Fgf8* were inhibited. (A,C) Inhibition of *Xag* (A) and *Xagr2* (C) mRNA translation by anti-sense morpholinos elicits reduction of the telencephalon (see enlarged images in the upper row, black arrow), otic vesicles (yellow arrowhead) and eyes (red arrowhead for *Xag* downregulation). (A′,C′) Overlays of the white light and fluorescent images of embryos shown in panels A and C demonstrate the distribution of cells containing injected MO mixed with a FLD tracer. (B,D) Rescue of anatomical abnormalities by co-injection of *Xag* and *Xagr2* mRNAs with *Xag* and *Xagr2* morpholinos to these genes. (B′,D′) Overlays of the white light and fluorescent images of embryos shown in panels B and D demonstrate the distribution of cells containing the injected MO mixed with a FLD tracer.

When *Xag* MO was injected, we observed head malformations, including partial reduction of the telencephalon, olfactory pit, otic vesicle, eye and branchial arches (110/120) (Fig. 5A). These malformations resembled those observed in experiments with downregulated Ras-dva1 (Tereshina et al., 2006). In contrast, injections of *Xagr2* MO primarily caused a reduction in the telencephalon, olfactory pits and otic vesicles (85/95), while branchial arches and eyes were reduced only in a small portion of embryos (9/95) (Fig. 5C). In spite of this difference, which could likely be explained by broader expression domains of *Xag* than *Xagr2* (Novoselov et al., 2003) or by some functional difference between these two proteins, these results confirm the importance of both Xag and Xagr2 for the forebrain development.

To test specificity of *Xag* and *Xagr2* MO effects, rescue experiments were performed by co-injecting these MOs with *Xag* or *Xagr2* mRNAs deprived of the MO target sites. As a result, a 50% rescue of the abnormalities induced by the MO injected alone was observed in both cases (35/72 and 28/55, respectively) (Fig. 5B,D). In addition, co-injection of same MOs with *Ras-dva1* mRNA did not cause any rescue (0/64) (data not shown). Notably, co-injections of *Xag* mRNA or *Xagr2* mRNA with *Ras-dva1* MO resulted in a 50% rescue (34/70 and 33/65) of abnormalities elicited by this MO injected alone (20/23 and 25/28) (Fig. 3G,I). This result is consistent with the hypothesis that positions Ras-dva1 upstream of Agrs in the regulatory pathway.

### Agrs, Fgf8 and Ras-dva1 regulate expression of each other

**Fig. 6.**
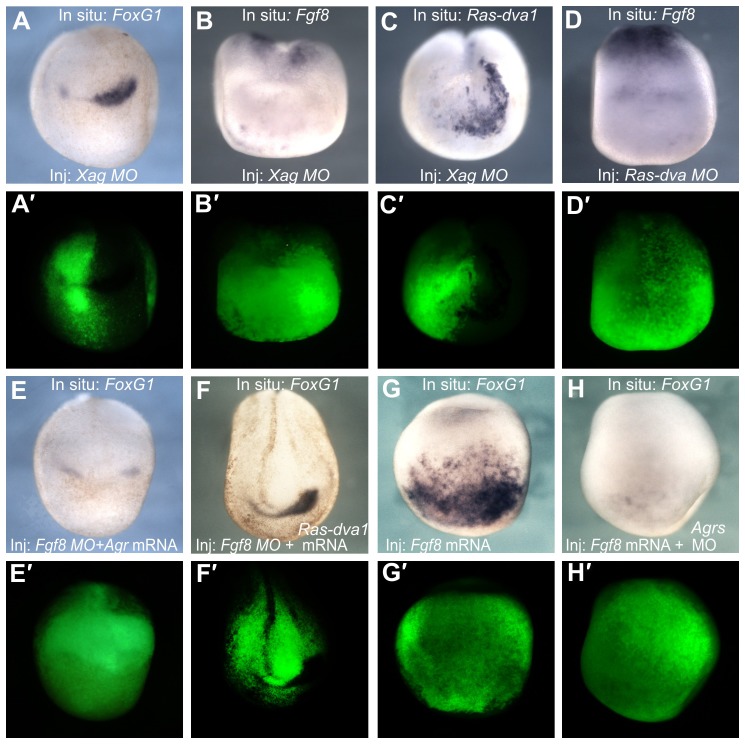
Agrs, Fgf8 and Ras-dva1 regulate expression of each other. (A–C) Injections of *Xag* and *Xagr2* MO elicit inhibition of *FoxG1*, *Fgf8* and *Ras-dva1* expression in the anterior ectoderm of midneurula embryos. (D) Injections of *Ras-dva* MO elicit inhibition of *Fgf8* expression in the anterior ectoderm of midneurula embryos. (E,F) Co-injection of *Agr* mRNA (equimolar mixture of *Xag1*, *Xag2*, *Xagr2A* and *Xagr2B* mRNAs) or *Ras-dva1* mRNA is unable to prevent the inhibitory influence of *Fgf8* MO on *FoxG1* expression. (G) Injection of *Fgf8a* mRNA elicits massive ectopic expression of *FoxG1* in the anterior ectoderm. (H) Induction of *FoxG1* expression elicited by ectopic Fgf8a is suppressed by co-injection of *Agrs* MO (a mixture of *Xag* and *Xagr2* MO). (A′–H′) Fluorescent image of the embryo shown in panels A–H demonstrates the distribution of injected cells labeled by the co-injected FLD tracer.

Because downregulation of *Agrs* caused a reduction in the forebrain structures, one may suppose that this effect could be due to the inhibition of some genes that regulate development of these structures. Indeed, the expression of the telencephalic regulators *FoxG1* and *Fgf8* was reduced in midneurula stage embryos injected with *Xag* MO (34/37 and 32/37) (Fig. 6A,B). Interestingly, inhibition of *Ras-dva1* expression was also observed in these embryos (30/36) (Fig. 6C). Given that downregulation of *Ras-dva1* inhibited *Agr* expression (Fig. 3), this result indicates a regulatory feedback loop between *Ras-dva1* and *Agrs*.

Speculating on a possible mechanism of such a feedback loop, one may suppose that one of its important components could be Fgf8, because it is upregulated by Agrs and since its activity is critical for the expression of *Ras-dva1*. If so, one may predict that the inhibition of Ras-dva1 functioning should result in downregulation of the *Fgf8* expression. Indeed, we observed just this effect in embryos microinjected with *Ras-dva1* MO (Fig. 6D).

Given that Agrs stimulate *FoxG1* expression, which in turn is upregulated by Fgf8 signaling (Danesin and Houart, 2012), one may suppose that the observed downregulation of *FoxG1* in embryos with inhibited Agrs could be caused by cessation of *Fgf8* expression in the ANB cells. In addition, Agrs might influence *FoxG1* expression directly. However, no rescue of the *FoxG1* abnormal phenotype generated by the *Fgf8* MO was observed in embryos co-injected with mRNA *Agrs* (2/37) or *Ras-dva1* (0/24) (Fig. 6E,F). Therefore, we concluded that Agrs upregulate *FoxG1* expression via stimulation of *Fgf8* expression.

As we have shown previously, an inhibition of *Ras-dva1* translation can interrupt upregulation of *FoxG1* expression by the ectopic Fgf8a (Tereshina et al., 2006). Together with the data described above, this result indirectly indicates that besides its stimulating influence on *Fgf8* expression, Agrs might be important for the functioning of the Fgf8 signaling per se. In that case one may predict that downregulation of Agrs, similarly to the inhibition of Ras-dva1, will interrupt the Fgf8a-stimulated induction of the *FoxG1* expression. Indeed, we observed just this effect when *Fgf8a* mRNA was co-injected with *Agrs* MO (44/46) (Fig. 6G,H).

### Ras-dva1-mediated Fgf8 signaling induces expression of *Agrs* through upregulation of *Otx2*

We have shown previously that the homeodomain transcription factor Otx2 can directly activate the expression of *Ras-dva1*, while the activity of Ras-dva1 in turn is essential for the expression of *Otx2* (Tereshina et al., 2006). Given that Otx2 is also an activator of *Agrs* expression (Ermakova et al., 1999; Gammill and Sive, 1997; Gammill and Sive, 2000), one may suppose that Ras-dva1-mediated Fgf8 signaling induces expression of *Agrs* by upregulating *Otx2*.

**Fig. 7.**
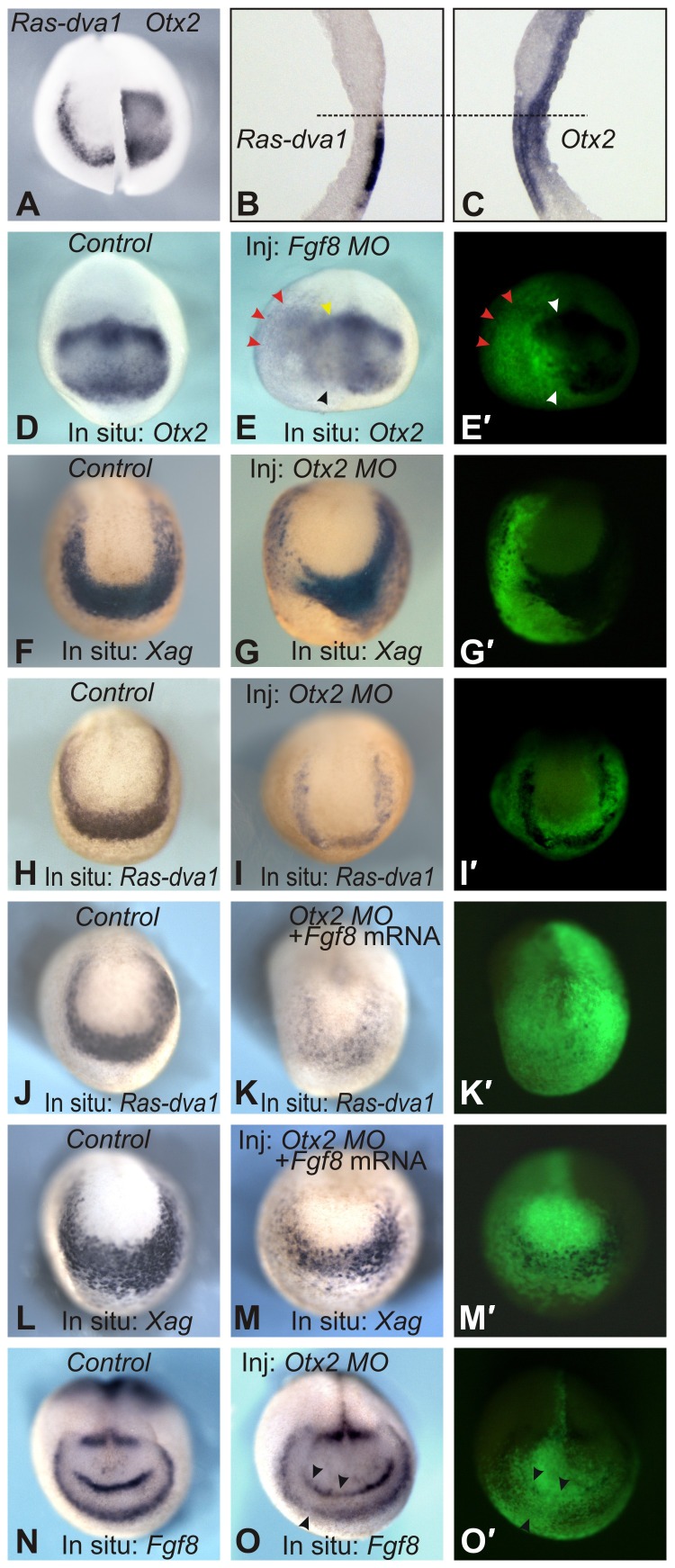
Ras-dva1-mediated Fgf8 signaling induces expression of *Agrs* by upregulating *Otx2*. (A–C) *Otx2* is co-expressed with *Ras-dva1* in cells of the outer layer of the anterior ectoderm. In situ hybridization on the left and right halves of the entire midneurula embryo and on sagittal vibratome sections was performed as described in the legends to Figs 1 and 2. (D,E) Inhibition of *Fgf8* mRNA translation by an *Fgf8* MO elicits partial inhibition of *Otx2* expression and lateral and posterior expansion of the expression domain (red arrowheads). Yellow and black arrowheads indicate a reduction of high *Otx2* expression in the presumptive midbrain and cement gland, respectively. (F,G,H,I) Inhibition of *Otx2* mRNA translation by an *Otx2* MO inhibits *Xag* and *Ras-dva1* expression. (J,K,L,M) Co-injection of *Fgf8a* mRNA is unable to prevent the inhibitory influence of the *Otx2* MO on *Xag* and *Ras-dva1* expression. (N,O) Inhibition of *Otx2* mRNA translation by the *Otx2* MO inhibits *Fgf8* expression (black arrows). (E′,G′,I′,K′,M′,O′) Fluorescent images of embryos shown in panels E,G,I,K,M,O demonstrate distribution of injected cells labeled by the co-injected FLD tracer.

To verify this, we first analyzed the expression pattern of *Otx2* in embryonic sections and confirmed that this gene is indeed co-expressed with *Ras-dva1* and *Agrs* in cells in the outer layer of the anterior ectoderm (compare Fig. 7A–C with Fig. 2C1–C4). Furthermore, to test whether endogenous Fgf8 signaling is essential for *Otx2* expression, we injected embryos with an *Fgf8* MO. As a result, a reduction in *Otx2* expression was observed in regions where this gene is expressed most intensely in normal development, i.e. in cells of the presumptive cement gland (27/34) and mesencephalon (23/34) (Fig. 7D,E, black arrowheads). However, the reduction in *Otx2* expression was not complete and a low level of expression was still observed in all cases examined. In addition, a characteristic feature of embryos injected with the Fgf8 MO was a posterior and lateral expansion of the low level of *Otx2* expression (Fig. 7E, red arrowheads). This result indicates that Fgf8 activity is essential both for the enhancement of *Otx2* expression in cells in the presumptive midbrain and cement gland and for restricting expression within these domains. Consistently, at later stages, the inhibition of *Otx2* expression in embryos injected with an *Fgf8* MO correlated with a reduction in the cement gland (17/30) and forebrain (24/30), i.e. anatomical structures whose development is controlled by Otx2 (supplementary material Fig. S4A,B). Notably, similar abnormalities were seen in embryos injected with *Agr* (20/32 and 26/32) or *Ras-dva1* (17/27 and 24/27) MOs (supplementary material Fig. S4C,D and data not shown).

Interestingly, inhibition of *Otx2* expression accompanied by expansion of the low expression domains was also characteristic for embryos injected with *Fgf8a* RNA (24/32) (supplementary material Fig. S4E,F). However, in contrast to the embryos injected with the *Fgf8* MO, the *Fgf8a* mRNA injected embryos demonstrated an expanded area of low *Otx2* expression and stripe-like zones of enhanced expression at the periphery of the injected territories (15/32) (supplementary material Fig. S4E,F, arrowheads). Given that Otx2 is the transcriptional activator of *Agrs* and *Ras-dva1*, this result is consistent with our data demonstrating that the last two genes were also strongly expressed at the periphery of cell clones bearing the exogenous *Fgf8a* mRNA (Fig. 4B,C).

To verify that Otx2 activity is indeed critical for *Ras-dva1* and *Agr* expression by another method, we analyzed expression of these genes in embryos injected with *Otx2* MO. As a result, we observed a significant downregulation of both genes in the injected embryos (21/28 and 24/30) (Fig. 7F–I). Importantly, these effects *Otx2* MO could not be rescued by co-injection of *Fgf8* mRNA (4/27), which, if injected alone, readily induced the expression of *Ras-dva1* and *Agrs* (compare Fig. 4A–C with Fig. 7J–M). Consistently, *Otx2* mRNA was able to partially rescue cessation of *Xag* expression elicited by *Fgf8* MO (35/42) (supplementary material Fig. S4G,H). By contrast, *Ras-dva1* mRNA could not cause similar effect (0/42) (supplementary material Fig. S4I), which indicates the necessity of Fgf8 signaling for the Ras-dva1 activity. In turn, an essential role of Fgf8 for the *FoxG1* expression is demonstrated by the fact that neither Ras-dva1, nor Otx2 mRNA were able to rescue *FoxG1* expression when these mRNA were co-injected with *Fgf8* MO (0/35 and 0/32, respectively) (Fig. 6F; supplementary material Fig. S4J,K).

At the same time, we found that Otx2 activity is important for maintaining *Fgf8* expression as a reduction in expression was observed in embryos injected with an *Otx2* MO (17/25) (Fig. 7N,O). This result confirms the participation of Otx2 in the signaling feedback loop between ANB cells and the adjacent anterior non-neural ectoderm.

## DISCUSSION

### Ras-dva1 regulates propagation of Fgf8 signaling in cells in the outer layer of the anterior non-neural ectoderm

As we have shown previously, small GTPase Ras-dva1 controls the telencephalic development in *Xenopus laevis* embryos by regulating propagation of Fgf8 signaling produced by ANB cells (Tereshina et al., 2006). The following data obtained in the present work confirm the non-autonomous character of this mechanism and demonstrate that it is based on an exchange of Fgf8 and Agrs signals between the neural and non-neural compartments at the ANB.

First, *Ras-dva1* is expressed exclusively in cells in the outer layer of the non-neural ectoderm bordering the ANB and, thus, in principal could not regulate expression of the telencephalic genes in the inner layer by a cell autonomous mechanism. Consistently, targeted downregulation of *Ras-dva1* elicits inhibition of expression of *FoxG1* and *Fgf8* in the telencephalic primordium in the inner layer of the anterior neural plate and reduction of the telencephalon. However, these abnormalities could be eliminated by co-injection of *Agr* mRNA. Second, blocking endogenous *Fgf8* mRNA translation prevents *Ras-dva1* and *Agrs* expression in the adjacent Fgf8-non-expressing cells in the outer layer. In agreement with this, the expression of *Ras-dva1* and *Agrs* can be induced by exogenous Fgf8, and this effect can be interrupted by blocking *Ras-dva1* mRNA translation. Third, downregulation of *Agrs* elicits inhibition of *FoxG1* and *Fgf8* expression and reduction of the telencephalon, i.e. the effects similar to those elicited by the *Ras-dva1* MO. However, neither Xagr, nor Ras-dva1 mRNA were able to prevent inhibition of *FoxG1* expression caused by *Fgf8* MO. The latter results demonstrate an absolute necessity of Fgf8 for the induction of *FoxG1*. At the same time, an ability of *Agrs* MO to interrupt *FoxG1* induction by Fgf8a indicates that Agrs, besides their influence upon Fgf8 expression, regulate Fgf8 signaling per se. Finally, the data in our previous (Tereshina et al., 2006) and present studies indicate that *Ras-dva1* expression in the outer, non-neural layer of the anterior neural fold is directly upregulated by the transcription factor Otx2. Otx2 also operates as an activator of *Agrs*, and in turn *Otx2* gene expression is activated by Fgf8 signaling transmitted by Ras-dva1 in the non-neural cells adjacent to the ANB (Ermakova et al., 1999; Gammill and Sive, 1997; Gammill and Sive, 2000).

**Fig. 8.**
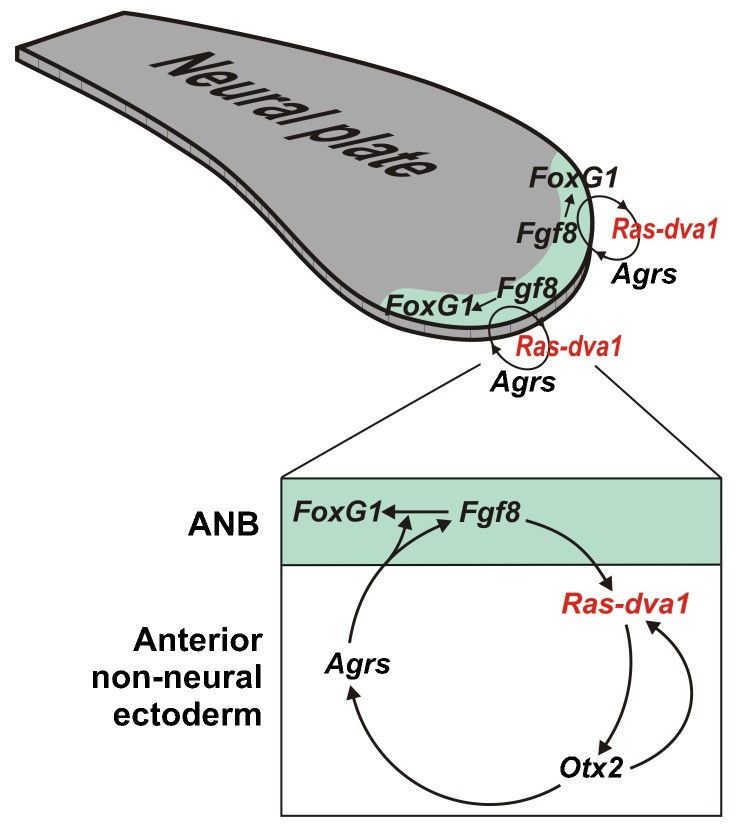
Model for the Fgf8- and Agrs-based signal exchange between neural and non-neural cells at the anterior neural plate border. Fgf8 produced by the ANB cells induces the expression of *Agrs* in cells adjacent to the anterior non-neural ectoderm by mediating Ras-dva1 and Otx2. In turn, Agrs secreted by cells in the anterior non-neural ectoderm promote forebrain development through stimulation of both *Fgf8* gene expression and Fgf8 protein signaling in the ANB cells.

Together all these data suggest a model in which Fgf8 produced by the ANB cells induces *Agrs* expression in the cells adjacent to the anterior non-neural ectoderm by mediating Ras-dva1 and Otx2. In turn, Agrs secreted by cells in the anterior non-neural ectoderm regulate telencephalic development as through stimulation of *Fgf8* expression in the ANB cells, as well as by promotion of Fgf8 signaling per se (Fig. 8).

The importance of ANB as a signaling center that regulates patterning of the rostral forebrain rudiment has been studied thoroughly in zebrafish and mice (Cavodeassi and Houart, 2012; Houart et al., 2002; Houart et al., 1998). However, the role of possible signal exchanges between cells in the ANB and the adjacent non-neural ectoderm was not studied. To our knowledge, only data on the role of BMP signals generated by cells of the non-neural ectoderm and regulating the anterior neural plate patterning have been published (Barth et al., 1999; Houart et al., 2002; Shimogori et al., 2004). Our finding, which demonstrates that the pattern of Agr secreted proteins is necessary, is another example of such a signal produced by cells in the non-neural ectoderm.

### Agrs control both forebrain development and body appendage regeneration

We demonstrate here that the activities of Xag1/Xag2 and Xagr2A/Xagr2B promote expression of at least two ANB regulators, *Fgf8* and *FoxG1*, and are necessary for forebrain development. These results are consistent with the data from other authors, who showed that ectopic Xag2 was able to induce several anterior markers in the embryonic ectoderm (Aberger et al., 1998). On the other hand, the newt Xagr2A/Xagr2B homolog was reported to be a key player during limb regeneration in adult salamanders (Kumar et al., 2007). In agreement with this, we have recently demonstrated that Xag1/2 and Xagr2A/Xagr2B are involved in the regeneration of the tail and hindlimb bud in *Xenopus laevis* tadpoles (Ivanova et al., 2013).

Given these data, an important future study is to compare the molecular mechanisms that regulate *Agrs* expression during body appendage regeneration and forebrain development. In particular, an area of interest for these studies is whether Fgf8, which participates in both these processes (Christen and Slack, 1997), might also be involved in regulating *Agrs* expression during tail and limb bud regeneration in *Xenopus* tadpoles. Furthermore, given that Ras-dva1 was shown to be involved in *Agrs* induction by Fgf8 in the anterior non-neural ectoderm, an important question is whether this small GTPase plays the same role during the regeneration of body appendages.

Another critical issue concerns the molecular mechanism of Agrs function. Importantly, evidence suggests that in contrast to other PDIs, Agrs act extracellularly (Kumar et al., 2011; Vanderlaag et al., 2010). Therefore, one may hypothesize that Agrs by themselves might play a role as signaling factors that interact with some as-yet unidentified receptors in cells of the inner layer. In particular, during regeneration of the salamander limb bud, Agr2 homolog regulates the expression of regenerating blastema-specific genes via binds to the membrane-anchored receptor Prod1 (Blassberg et al., 2011; Kumar et al., 2011; Kumar et al., 2007).

Alternatively, as Agrs belong to the superfamily of protein disulfide isomerases (PDI), which modulate folding of other proteins (Hatahet and Ruddock, 2009; Persson et al., 2005), they might regulate the folding of some extracellular proteins in the intercellular space. Such a supposition is supported by our data demonstrating ability of Agrs to regulate Fgf8 signaling per se, besides influencing *Fgf8* expression. Accordingly, one may further suppose that by this way Agrs might regulate folding of Fgf8, or/and its receptor(s), or/and their co-factors.

An important question for further study is to understand which of these molecular mechanisms is implemented during forebrain development and body appendage regeneration.

## Supplementary Material

Supplementary Material
