# Supplementary material for: Ras-dva1 small GTPase regulates telencephalon development in Xenopus laevis embryos by controlling Fgf8 and Agr signaling at the anterior border of the neural plate
Source: Biol Open. 2014 Feb 25;3(3):192–203. doi: 10.1242/bio.20147401 (PMC4001240; doi:10.1242/bio.20147401)
Supplement: Supplementary Material [file supp_3_3_192__index.html]

Ras-dva1 small GTPase regulates telencephalon development in Xenopus laevis embryos by controlling Fgf8 and Agr signaling at the anterior border of the neural plate — Supplementary Material 

# Ras-dva1 small GTPase regulates telencephalon development in *Xenopus laevis* embryos by controlling Fgf8 and Agr signaling at the anterior border of the neural plate

## bio.20147401 Supplementary Material

**Files in this Data Supplement:**

- Supplementary Material - Maria B. Tereshina et al. doi: 10.1242/bio.20147401
