# Supplementary material for: Ras-dva1 small GTPase regulates telencephalon development in Xenopus laevis embryos by controlling Fgf8 and Agr signaling at the anterior border of the neural plate
Source: Biol Open. 2014 Feb 25;3(3):192–203. doi: 10.1242/bio.20147401 (PMC4001240; doi:10.1242/bio.20147401)
Supplement: Supplementary Material [file supp_bio.20147401_bio.20147401-s1.pdf]

Biology Open (2014) 000, 1–9 doi:10.1242/bio.20147401

Biology Open

Supplementary Material

Maria B. Tereshina et al. doi: 10.1242/bio.20147401

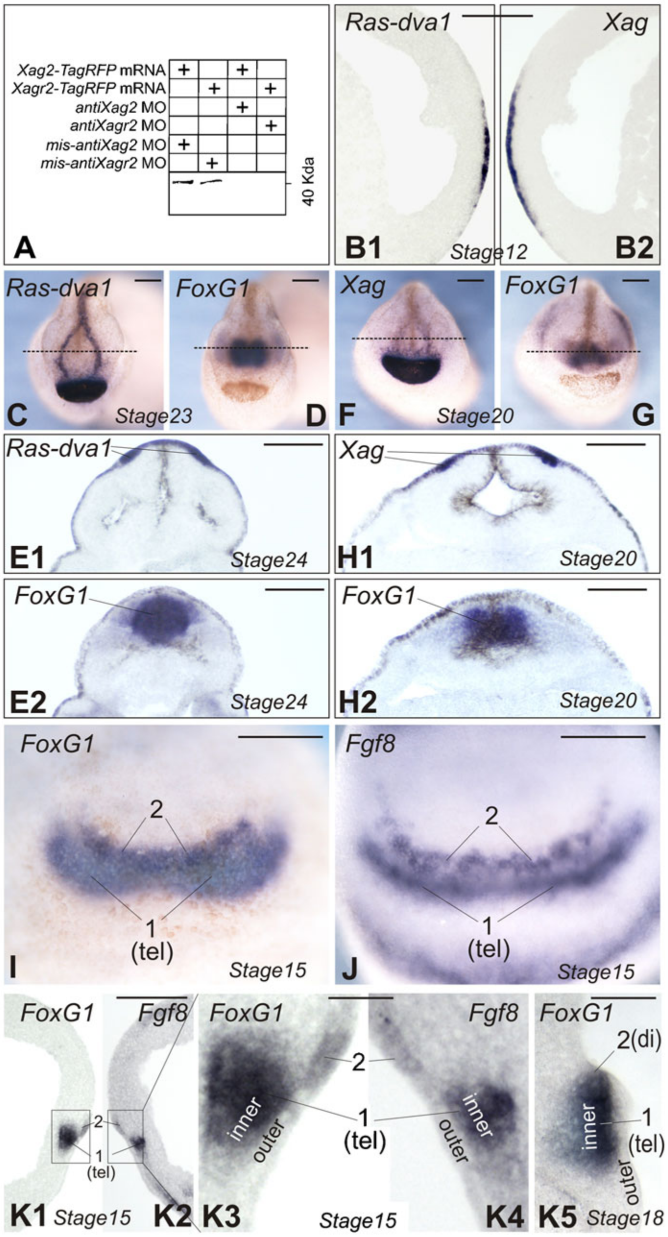

Fig. S1. Testing of *Xag* and *Xagr2* MOs efficiency and analysis of *Ras-dva1*, *Xag*, *FoxG1* and *Fgf8* expression. (A) *Xag2-TagRFP* and *Xagr2-TagRFP* mRNA were injected in dorsal blastomeres of 8-cell embryos (100 pg/blastomere) either alone or in a mixture with the corresponding MO (8 nl of 0.4 mM water solution). The injected embryos were collected at the midneurula stage and analyzed for presence of Xag2-TagRFP and Xagr2-TagRFP proteins by Western blotting with an anti-tRFP antibody (see Materials and Methods for details). (B1,B2) At the late gastrula (stage 12), *Ras-dva1* and *Xag* are expressed exclusively in the outer layer of the anterior ectoderm. No expression is in the inner layer, in which *FoxG1* and *Fgf8* begin to be expressed with the onset of neurulation. Adjacent vibratome sagittal sections of the same embryo were hybridized separately with *Ras-dva1* (left section) or *Xag* (right section) probe. Anterior sides face each other, dorsal sides up. (C,D) Embryos at the tailbud stage (stage 23) hybridized in whole-mount with probes to *Ras-dva1* and *FoxG1*, respectively. Dashed lines indicate approximate levels of sections shown in panels E1 and E2. Anterior view, dorsal sides up. (E1,E2) Adjacent vibratom frontal sections of the same stage 23 embryo hybridized separately with *Ras-dva1* or *FoxG1* probe (see approximate levels of sections in panels C and D). Anterior up. (F,G) Embryos at the tailbud stage (stage 20) hybridized in whole-mount with probes to *Xag* and *FoxG1*, respectively. Dashed lines indicate approximate levels of sections shown in panels H1 and H2. Anterior view, dorsal sides up. (H1,H2) Adjacent frontal sections of the same stage 20 embryo hybridized separately with *Xag* or *FoxG1* probe (see approximate levels of sections in panels C and D). Anterior up. (I,J) Expression of *FoxG1* and *Fgf8* in presumptive telencephalic (inner layer, zone 1) and non-telencephalic (outer layer, zone 2) cells as it is seen in the midneurula embryos hybridized in whole-mount. Anterior view, dorsal side up. (K1–K4) Expression of *FoxG1* and *Fgf8* revealed on adjacent sagittal sections of the same embryo at midneurula stage. Vibratom sections of the same embryo were hybridized separately with *FoxG1* or *Fgf8* probe. Anterior sides face each other, dorsal sides up. (K5) Expression of *FoxG1* in the same region as shown in panel K3 but at stage 18 (late neurula). Note that no expression is seen in the outer layer except few cells in posterior region of the expression spot. This region is in the internal surface of the anterior neural fold and further hives rise to the diencephalon. Scale bars: 200 μm (B1–K2), 40 μm (K3–K5).

Biology Open (2014) 000, 1–9 doi:10.1242/bio.20147401

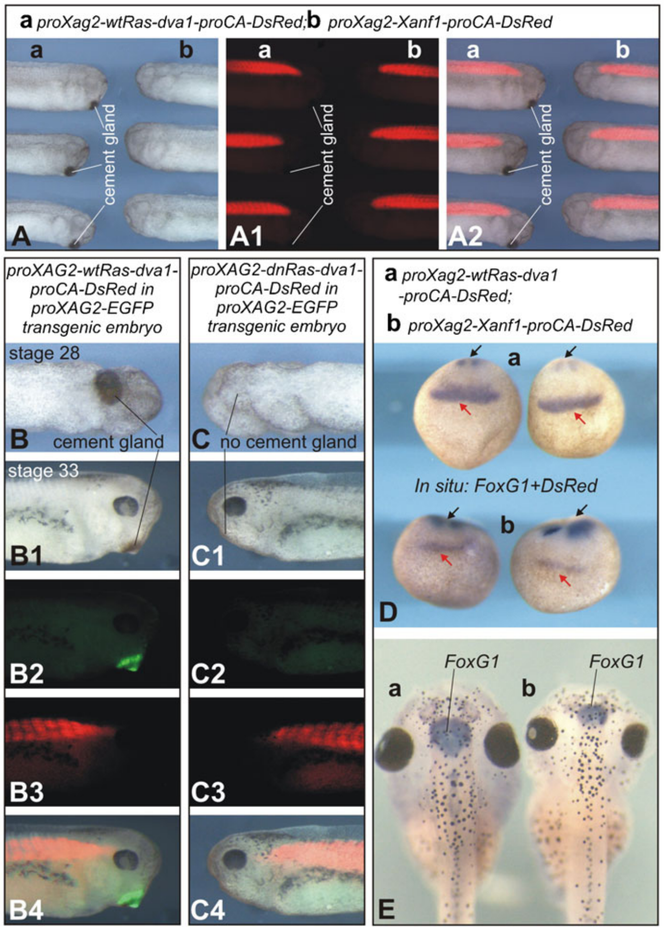

Fig. S2. Analysis by transgenic embryos of the effects of *Ras-dva1* downregulation. (A–A2) Whereas no cement gland inhibition is revealed in control transgenic embryos expressing wild-type Ras-dva1 under the control of Xag2 promoter (*proXag2-wtRas-dva1-proCA-DsRed* transgene), the cement glands are inhibited in embryos expressing *Xanf1* under the same promoter (*proXag2-Xanf1-proCA-DsRed* transgene). (A) Bright light, (A1) DsRed fluorescence, (A2) overlay of panels A and A1. (B–C4) Whereas no cement gland inhibition is seen in control embryo of transgenic line bearing proXag2-EGFP construct and transfected by *proXag2-wtRas-dva1-proCA-DsRed* (B–B4), the embryo of the same line but transfected by *proXAG2-dnRas-dva1-proCA-DsRed* construct has no cement gland (C–C4). (D) No inhibition of *FoxG1* expression is seen in the early neurula embryos bearing the control transgene *proXag2-wtRas-dva1-proCA-DsRed* (a). In contrast, a decrease of *FoxG1* expression is observed in embryos transfected with *proXAG2-Xanf1-proCA-DsRed*. Whole-mount in situ hybridization with probes to both *FoxG1* and *DsRed*. (E) Transgenic tadpole bearing the control *proXag2-wtRas-dva1-proCA-DsRed* construct has normal sized telencephalon marked by *FoxG1* expression (a). At the same time, a reduction of the telencephalon and *FoxG1* expression is seen in embryo bearing *proXAG2-Xanf1-proCA-DsRed* construct (b). Transgenic tadpoles were selected by revealing DsRed fluorescence and hybridized in whole-mount with the probe to *FoxG1*.

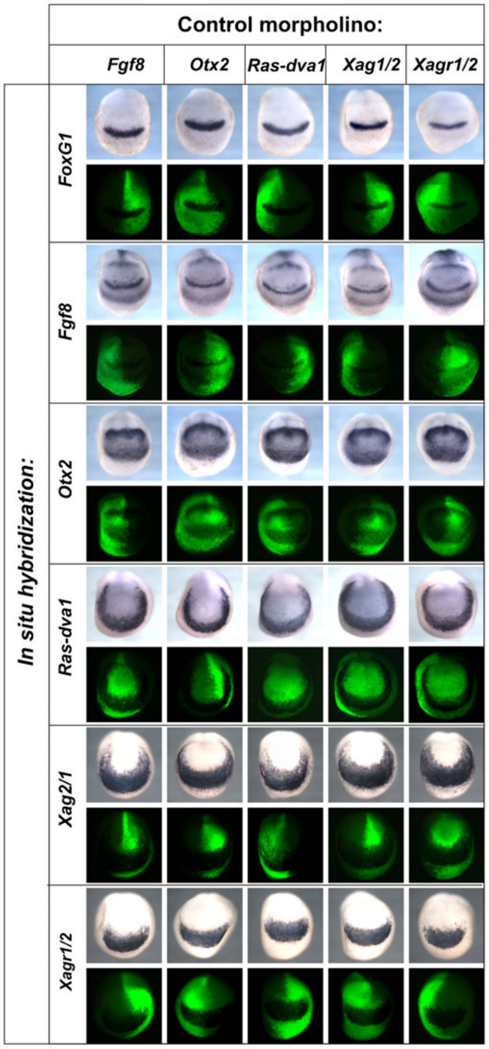

Fig. S3. Lack of effects of control morpholino oligonucleotides on expression of all genes studied in this work. Control MO (supplementary material Table S1) to all genes whose expression was inhibited by active MO were injected in concentration 1 mM (3–5 nl/blastomere) in one of the animal dorsal blastomeres at 8 blastomere stage in mixture with living tracer FLD. Injected embryos were collected at midneurula stage and hybridized in whole-mount with probes to all genes analyzed in this work.

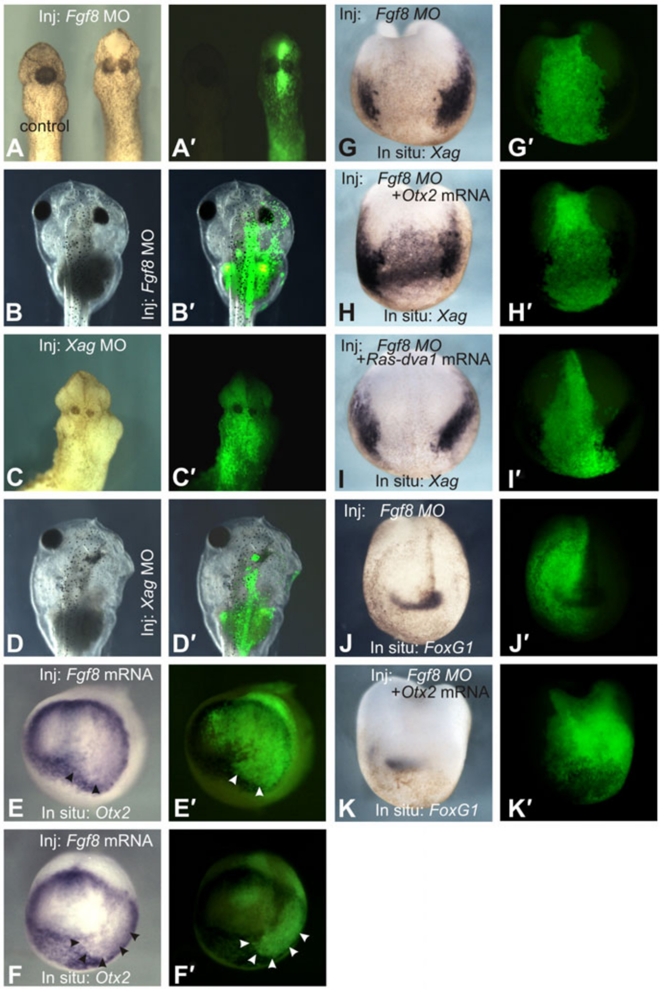

Fig. S4. Effects elicited by misexpression of *Fgf8*, *Otx2* and *Xag*. (A,B,C,D) Inhibition of *Fgf8a* and *Xag* mRNA translation by antisense morpholino oligonucleotides inhibit cement gland differentiation and cause a reduction in the telencephalon and eyes. (E,F) Examples of the midneurula stage embryos (anterior view with the dorsal side upward) injected with *Fgf8a* mRNA and hybridized in whole mount with a dig-labeled probe to *Otx2*. Note the expansion of the domains with low *Otx2* expression that are bordered along the injected areas by stripes of enhanced expression. Black and white arrowheads indicate the borders of the injected cell clones. (G,H) Whereas *Fgf8* MO injected alone results in the inhibition of *Xag* expression, co-injection of *Otx2* mRNA elicits rescue of *Xag* expression. (I) *Ras-dva1* mRNA co-injected with *Fgf8* MO cannot rescue *Xag* expression. (J,K) In contrast to *Xag*, co-injection of *Otx2* mRNA is unable to rescue *FoxG1* expression inhibited by Fgf8 MO.

Table S1. The morpholino oligonucleotides used in this work

mRNA	Morpholino	Control morpholino
Fgf8	5′-GGAGGTGATGTAGTTCATGTTGCTC (Fletcher et al., 2006)	5′-GCAGGGGATATAGTTGATGTTACTA
Otx2a/b	5′-GGTTGCTTGAGATAAGACATCATGC (Carron et al., 2005; Hyenne et al., 2005)	5′-GTTTTCTTGAAATAGGACATAATGT
Ras-dva1	5′-GTGAGATTGCGCTTTCTTTTGTCTG (Tereshina et al., 2006)	5′-GTGACATTGCTCTTTCTTTTGTGTT
Xag1/2	5′-TCTGTGGATGTCTTGCTCTTCCAGG	5′-TATGTGTATGTATTGCTGTTCAAGA
Xagr2A/B	5′-CAGTGCTTTACTCCAGAGGCAGGAG	5′-CAATGCTATACTGCAGATGCACGAT
